# Recommendations for the Management of Cardiomyopathy Mutation Carriers: Evidence, Doubts, and Intentions

**DOI:** 10.3390/jcm12144706

**Published:** 2023-07-15

**Authors:** José F. Couto, Elisabete Martins

**Affiliations:** 1 Faculty of Medicine, University of Porto, 4200-450 Porto, Portugal; ebernardes@med.up.pt; 2 Centro Hospitalar Universitário São João, Member of the European Reference Network for Rare, Low-Prevalence, or Complex Diseases of the Heart (ERN GUARD-Heart), 4200-319 Porto, Portugal; 3 Center for Health Technology and Services Research (CINTESIS@RISE), Faculty of Medicine, University of Porto, 4200-450 Porto, Portugal

**Keywords:** hypertrophic cardiomyopathy, dilated cardiomyopathy, arrhythmogenic cardiomyopathy, noncompaction

## Abstract

Cardiomyopathies may be hereditary and associated with a familial predilection. Morbidity and mortality can be caused by heart failure, sudden death, or arrhythmias. Sometimes these events are the first manifestations of cardiovascular disease. Hypertrophic cardiomyopathy and arrhythmogenic cardiomyopathy are perhaps most thoroughly studied in that context. Dilated cardiomyopathy, although most frequently of secondary etiology, has a significant familial cluster. Noncompaction of the left ventricle can sometimes be seen in healthy individuals and, in other instances, is associated with severe LV dysfunction. Genetic testing is of utmost importance, since it might allow for the identification of individuals carrying mutations predisposing them to these diseases. In addition, certain variants may benefit from tailored therapeutic regimens, and thus searching for a causal mutation can impact clinical practice and is recommended for all patients with HCM or ACM. Patients with DCM and positive family history should be included as well. Regular follow-ups are advised, even in those with negative phenotypes, because these disorders are often age dependent. During pregnancy and in the case of athletes, special consideration should be made as well. We intend to summarize the most current evidence regarding their management.

## 1. Introduction

The term cardiomyopathy refers to a complex and heterogenous group of conditions that present with functional and or structural myocardial anomalies without predisposing factors, such as volume/pressure overload or coronary atherosclerosis [1]. The genetic basis of these diseases has been the subject of interest for many authors. In the last couple of years, multiple studies regarding the management of patients with cardiomyopathies have been published. In this review, we intend to summarize the recommendations for the management of familial cardiomyopathies, namely, current indications for genetic testing in cardiomyopathies, and appropriate follow-up of patients carrying a mutation associated with a cardiomyopathy as well as pregnancy and sports restrictions.

**Figure 1:**
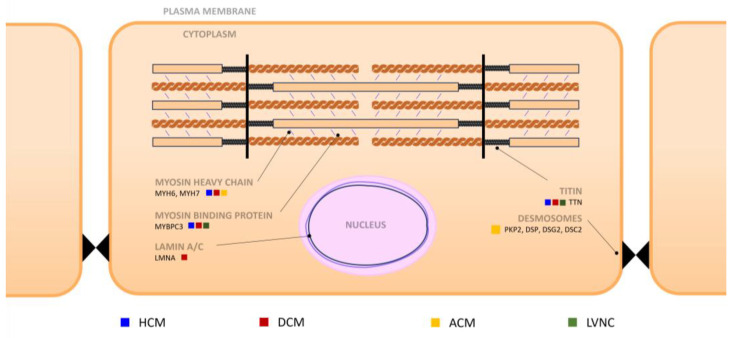
Cardiomyocyte showing the main proteins affected in genetic cardiomyopathies. Mutations involving proteins of the sarcomere are a cause of hypertrophic cardiomyopathy (HCM), the most frequent being myosine-binding protein C3 (MYBPC3) and myosine heavy chain 6 and 7 (MYH6, MYH7). The most common mutation in dilated cardiomyopathy (DCM) is titin (TTN). Lamin A/C (LMNA) of the nuclear envelope is also relevant. In arrhythmogenic cardiomyopathy (ACM), desmosome mutations are the most prevalent, including, plakophilin 2 (PKP2), desmoplakin (DSP), desmoglein 2 (DSG2), and desmocollin 2 (DSC2). In left ventricular noncompaction (LVNC), either MYBPC3 or TTN can be affected.

Most cardiomyopathies tend to present in adulthood and have autosomal dominant inheritance. In the pediatric age group, other patterns of transmission are more relevant. Autosomal recessive, X-linked, or mitochondrial inheritance appear at higher rates than in adults [2,3]. Given that a child may be the first individual in a family to be affected, of those without a family history, only a 1/3 can be attributed to de novo mutations. In the others, parents are asymptomatic or have not yet developed the disease [4,5]. The main genetic causes of each cardiomyopathy can be seen in Figure 1.

## 2. Methods

We reviewed the available literature on the PubMed database, using terms such as “genetics/cardiomyopathies”, “Cardiomyopathy, Hypertrophic”, “Cardiomyopathy, Dilated”, “Arrhythmogenic Right Ventricular Dysplasia”, and “Isolated Noncompaction of the Ventricular Myocardium”, up until the end of February 2023. To garner specific information regarding “epidemiology”, “genetics”, “pregnancy”, “athletes”, and “sudden death”, those terms were added to the search. In this narrative review, we included case reports, observational studies, clinical trials, and systematic reviews according to what we deem to be most pertinent. In addition, we analyzed various clinical guidelines from international scientific societies, including the European Society of Cardiology, the American Heart Association, and the American Heart Failure Society.

## 3. Hypertrophic Cardiomyopathy

The hallmark of hypertrophic cardiomyopathy (HCM) is the thickening of the left ventricular wall. Besides this imaging finding, electrocardiographic abnormalities, fibrotic infiltrates in the myocardium, as well as valvular and coronary artery anomalies may be present [6].

Usually, HCM has an autosomal dominant mode of inheritance and, in most cases, involves sarcomere proteins. In some instances, heredity may be recessive, appear de novo, or have low penetrance, hindering the recognition of a familial predisposition [7].

A vast spectrum of clinical findings has been described. HCM constitutes one of the main causes of sudden death in young people. On the other hand, it may lead to severe left ventricular function impairment and cause heart failure. Given its highly complex clinical manifestations, establishing a prognosis can present an intricate dilemma [6].

### 3.1. Prevalence

HCM is the most prevalent genetic cardiomyopathy. Still, it is estimated that most individuals carrying a mutation associated with HCM are not identified. In the general population, the prevalence of HCM is thought to be 0.2%. Estimates may vary, depending on whether only clinically evident disease or asymptomatic carriers are considered. In addition, population characteristics such as age may be relevant [8]. Some studies in the United States have reasonably predicted the true prevalence value to be between 1:200 to 1:500 people [9]. Women and African Americans tend to be more frequently underdiagnosed or diagnosed later than men and non-African Americans [10,11]. Moreover, age seems to be a relevant aspect in the development of the condition, since time is needed for the myocardium to become hypertrophic and thus cause symptoms [12].

### 3.2. Associated Genes

In up to 60% of cases, a disease-causing mutation is identified. Nowadays, regular genetic testing involves at least eight sarcomere or sarcomere-related genes: MYH7 (cardiac beta-myosin heavy chain), MYBPC3 (cardiac myosin binding protein C3), TNNT2 (cardiac troponin T), TNNI3 (cardiac troponin I), TPM1 (alpha-tropomyosin), ACTC (encoding cardiac actin), and MYL2 and MYL3 (myosin light chains). Most often than not, these mutations are missense, although loss and gain of function are possible [7,13].

Consequently, a significant percentage of HCM cases remains without a definitive genetic cause. Within a subset of individuals, neither a genetic cause nor a familial predisposition occurs, meaning that other genes or pathophysiological mechanisms of HCM have yet to be discovered [14].

### 3.3. Diagnosis and Disease Mechanism

Classical descriptions of the hypertrophy observed in HCM refer to asymmetrical growth of the interventricular septum. Other less frequent forms affect the free wall, the anterolateral wall of the left ventricle (LV), or the apex. Ultimately, the papillary muscles and the right ventricle may be involved [15].

All in all, a diagnosis in adults can be established by 2D echocardiography or cardiac magnetic resonance (CMR) once a maximal end-diastolic wall thickness (MEDWT) of 15 mm is found anywhere in the LV, seeing as though other causes of left ventricular hypertrophy (LVH) have been excluded. In patients with a family history or positive genetic testing, less restrictive diagnostic criteria of 13 or 14 mm can be used [16,17].

In children, the measurement of the LV dimension must be adjusted for, among other things, age and size. Thus, a z-score (number of standard deviations from the mean population) adjusted for a body surface greater than two is, usually, considered diagnostic. Yet, the American guidelines only consider that threshold adequate in cases with a definite family history or positive genetic testing and require a z-score ≥ 2.5 in asymptomatic children or negative family history [16,17].

## 4. Dilated Cardiomyopathy

Dilated cardiomyopathy (DCM) is the most frequent indication for cardiac transplantation. It can be defined by the presence of an enlarged and poorly contractile LV. Nonetheless, circumstances that could result in severe LV impairment such as significant coronary artery disease or abnormal loading conditions must be excluded to meet DCM criteria [18,19].

Therefore, a rigorous differential diagnosis of ischemic heart disease must be completed before safely establishing a diagnosis. In that case, angiography (or computed tomography coronary angiography) can show stenosis of the coronary vessels. Cardiovascular magnetic resonance can be of help, showing late gadolinium enhancement (LGE) compatible with previous myocardial infarction [20,21].

In addition, the same patterns of cardiac remodeling can appear in different nonischemic DCM or as a maladaptive response to environmental factors, so those situations ought to be ruled out. Toxins such as alcohol, amphetamines, or cocaine; some infections; and metabolic and inflammatory factors are potential etiologies of DCM [22].

Some unique yet relevant signs should raise suspicion of a multisystemic disease. A few examples of those constitute the presence of a unique pigmentation of the skin and scars, seen in hemochromatosis; the presence of muscle weakness or abnormal gait, suggesting a neuromuscular disease; and neurosensorial deafness, suggesting a mitochondrial disease [23].

### 4.1. Prevalence

The most up-to-date assessments predict DCM prevalence to be around 1:250. That is to say, DCM is no less common than HCM. When at least two individuals in the same family, either first- or second-degree, develop DCM without a clear etiology, it is defined as familial dilated cardiomyopathy (FDM) [24]. Sporadic forms of DCM tend to be more frequent than FDM. Once first-degree relatives of DCM patients are studied, FDM is diagnosed in 20–35% of the patients [25].

As seen with HCM, the recognition of FDM can be thwarted by incomplete pedigree assessment, the occurrence of de novo mutations, age-dependent phenotypes, or incomplete penetrance of existing mutations [12].

### 4.2. Associated Genes

DCM has been associated with nearly 40 different genes. Mutations related to the cytoskeletal complex, proteins of the nuclear envelope, sarcomere, ion channels, and transcription factors have been reported; however, up to 25% of all cases involve titin (TTN). Regarding the cytoskeletal complex proteins, the dystrophin gene is commonly impaired. This gene is, simultaneously, the cause of Duchenne muscular dystrophy (DMD). Desmin (DES) is related to the myocardium’s cytoskeleton architecture but also to the skeleton and smooth muscle. DES mutations have been reported in patients with skeletal or myocardial myopathies [26]. Furthermore, lamin A/C (LMNA) proteins of the nuclear envelope are implicated, having been found in individuals that develop earlier conduction system disease and have higher sudden cardiac death risk. Mutations in ion channels such as sodium channel gene SCN5A, also in Brugada syndrome or in familial Long QT Syndrome, have been reported as well [12,19].

RBM20 is an RNA binding protein. Autosomal dominant mutations have been described, typically associated with early onset disease and with a more severe clinical presentation [27].

Of all cases of FDM, up to 10% will carry a heterozygous mutation of a sarcomere gene, such as an MYH7 chain or ACTC. This means that FDM and HCM share some genetic causes. Currently, it has been proposed that in HCM, force generation is impaired, affecting the movement of contractile proteins, whereas in FDM, the mechanisms for relaying contractile force to the z-disks/cell membrane are altered [12].

Lastly, the interplay of multiple genes, for example due to digenetic inheritance, could significantly impact the severity of the phenotype. Recently it has been reported that a patient carrying both TTN and RBM20 mutations had an earlier and more severe onset of disease [28].

### 4.3. Diagnosis and Disease Mechanism

The proposed mechanism of DCM has long been understood to be the dilation of the LV due to fibrosis and other remodeling processes. Set structural changes result in pump failure, namely, decreased cardiac output and impaired ventricular filling with increased end-diastolic ventricular pressure. In response, various systemic compensatory adjustments arise. Often the diseases will slowly, but definitely, lead to heart failure [24].

Diagnostic criteria include LV or biventricular dysfunction and dilation, with an end-diastolic volume or diameter with a z-score > 2. Body surface area, age, and ejection fraction are frequent confounders to adjust for [24]. A similar entity without significant heart chamber dilation has been defined as hypokinetic non-dilated cardiomyopathy [29].

In some instances, to clarify the etiology, cardiac resonance imaging can be useful, since the detention of fibrosis and edema can point to an inflammatory cause, though with residual sensitivity. Ultimately, ECG is usually unnoteworthy, though repolarization anomalies, left bundle branch block, or auriculoventricular conduction prolongation can occur [24].

Morbidity and mortality in DCM are attributed to the development of heart failure or are consequences of a fatal arrhythmia [24].

## 5. Arrhythmogenic Cardiomyopathy

Arrhythmogenic cardiomyopathy (ACM) is an inherited disease with autosomal dominant traits frequently associated with desmosome proteins. It affects predominantly the right ventricle, where cardiomyocytes are replaced by the fibrotic extracellular matrix and adipocytes. Typically, these histological changes occur mainly on the right side of the heart, though left ventricle disease can happen, and, in limited cases, can be the predominant location [30,31].

Patients are predisposed to severe ventricular dysfunction and life-threatening arrhythmias. Accordingly, ACM is regarded as a leading cause of sudden death in young people and athletes [30,31]. Moreover, sudden cardiac death may be the only clinical manifestation of the disease. An Italian group found that ACM was undiagnosed in 20% of adults and athletes investigated for sudden death [32].

### 5.1. Prevalence

ACM prevalence in the general population could be 1:5000, making it a rare genetic disease [30,33].

Current estimates are that 50% of patients have a positive family history. However, familial predisposition is thought to be higher, which can be explained by incomplete penetrance and limited phenotypic expression [31].

### 5.2. Associated Genes

ACM is commonly associated with desmosomal gene mutations. Plakophilin 2 (PKP2) is the most frequent, followed by desmoplakin (DSP), desmoglein 2 (DSG2), and desmocollin 2 (DSC2). However, when searching for individuals meeting international diagnostic criteria for ACM, not more than half will meet these criteria. Unfortunately, even adding non-desmosomal mutations will only marginally help identify causal mutations [31].

Spatial transcriptomics studies have helped to corroborate some pathophysiological mechanisms of ACM. For example, it has been proven that PKP2 mutations are related to biventricular remodeling with fibro-fatty deposition in the myocardium, cardiomyocyte degeneration, and loss of desmosomes between cardiomyocytes [34].

One example of non-desmosomal mutations is transmembrane protein 43 (TMEM43), a nuclear envelope protein, with one specific mutation (TMEM43-p.S358L) known to cause a highly penetrant and lethal phenotype, although very rare and endemic in a specific region of Canada [35]. Other mutations in this gene have been described, and studies in zebrafish have shown that the loss and gain of function of this protein affects the morphology and function of the heart, similarly to what can be seen in ACM [36].

Integrin-linked kinase (ILK) is a serine/threonine protein kinase, with various mutations previously described in patients with ACM. Studies in zebrafish found that some mutations in this gene were associated with higher rates of premature death caused by heart failure [37].

Desmin (DES) is a protein of the cytoskeleton, and its function is related to the Z-discs in the sarcomeres but also desmosome plaque proteins. It has been associated with myofibrillar myopathies; thus, some have suggested that filament assembly defects could be involved in the pathophysiology of ACM [38].

### 5.3. Diagnosis and Disease Mechanism

ACM is defined by the presence of an “arrhythmogenic heart muscle” and is not caused by vascular, hypertensive, or ischemic disease [39].

The deposition of fibrofatty tissue initially occurs in the epicardium and then propagates toward the endocardium. Consequently, cardiac chamber walls become thinner to the point of aneurysmal dilation. The most common locations within the right ventricle are the sub-tricuspid and infundibular regions as well as the apex [31].

Initial clinical presentations are the onset of palpitations or even syncope with exertion coupled with electrocardiographic anomalies, for example, repolarization anomalies such as T-wave inversions, or depolarization anomalies such as a left bundle branch block. Long-standing patients may develop heart failure either limited to the right side or biventricular [31].

Currently, the gold standard for the diagnosis of ACM is histology, whether from biopsy or post-mortem autopsy. In fairness, imaging studies are more important in clinical practice. Echocardiography might show global ventricular dilation, systolic akinesia, dyskinesia, or diastolic bulging. CMR with gadolinium enhancement allows for detailed tissue characterization, non-invasively, making it the preferred method [39].

The European Society of Cardiology, in its Task Force Criteria for the diagnosis of ACM, proposed a mixture of ECG, imaging, clinical symptoms, family history, and genetic testing criteria to define the disease [40].

## 6. Left Ventricular Noncompaction

Left ventricular noncompaction (LVNC) may be a congenital disease with a genetic predisposition. Patients present with an excessively trabecular ventricle, forming recesses or sinusoids directly in communication with the left cavity due to abnormal embryogenesis [41,42].

Although sometimes isolated in some cases, LVNC can be associated with other cardiac anomalies, such as complex cyanotic congenital heart disease [42].

Imaging techniques, namely, 2D echocardiography, CMR, and angiography, can be employed to establish a diagnosis [41,42].

Heart failure, sudden death, and cardioembolism comprise the three main courses of the disease. LV systolic dysfunction can progress, and a heart transplant might be indicated. Besides that, thromboembolic complications are also important to be accounted for [42].

Though classical descriptions define LVNC as a congenital cardiomyopathy, “acquired” LVNC has been reported in patients with the onset of the disease later in life [43].

### 6.1. Prevalence

Generally, it is said that the prevalence of LVNC is unknown. Estimates depend greatly on population characteristics and the diagnostic criteria used, including imagological modality. Published evidence ranges widely, from 0.14% to 1.26% of patients referred to a given echocardiography laboratory [44].

### 6.2. Associated Genes

Several genes have been linked to LVNC and with various patterns of inheritance. Both autosomal dominant and recessive, also X-linked, and mitochondrial inheritance are possible. Most commonly, it is autosomal dominant and involves mitochondrial, cytoskeletal, sarcomere, or ion channel genes [45].

When studied, a mere 30% of probands revealed a positive family history [46]. Thus, in the majority of cases, LVNC is sporadic [47].

Taffazin (TAZ) or the G4.5 gene is a known genetic cause of LVNC. Particularly, this gene is identified in pediatric age, associated with myopathies such as Barth syndrome, but not in adults. Z-line protein, LIM domain binding protein 3 (LBD3), and lamin A/C protein genes have established a mutual genetic etiology between DCM and LVNC. An overlap with HCM also exists, with some sarcomere protein genes also being implied in LVNC [45].

LVNC in conjunction with congenital heart disease is often attributed to a mutation in the α-dystrobrevin gene codifying a cytoskeletal protein [42].

Mutations in the DES gene in the context of LVNC are rare; however, they tend to present simultaneously with neuromuscular and myocardial disease, having a poorer prognosis [48,49].

### 6.3. Diagnosis and Disease Mechanism

The literature regarding the median age of diagnosis of LVNC is extremely incoherent. In some studies, the authors have estimated it to be as little as 7 years old (from 11 months to 22 years), whereas in others, they calculated it to be 58 years (from 28 to 80 years). Currently, a growing number of patients are diagnosed in adulthood; however, when compared to the other cardiomyopathies mentioned above, LVNC is identified more prematurely [50,51].

Echocardiography in LVNC will show a two-layered myocardium in the LV. The layer adjacent to the epicardium is thin, whereas the one closest to the endocardium is thicker and replenished with prominent trabeculations and recesses. A Doppler sign can be found in set recesses, proving the connection to the main chamber of the LV [45].

**Figure 2:**
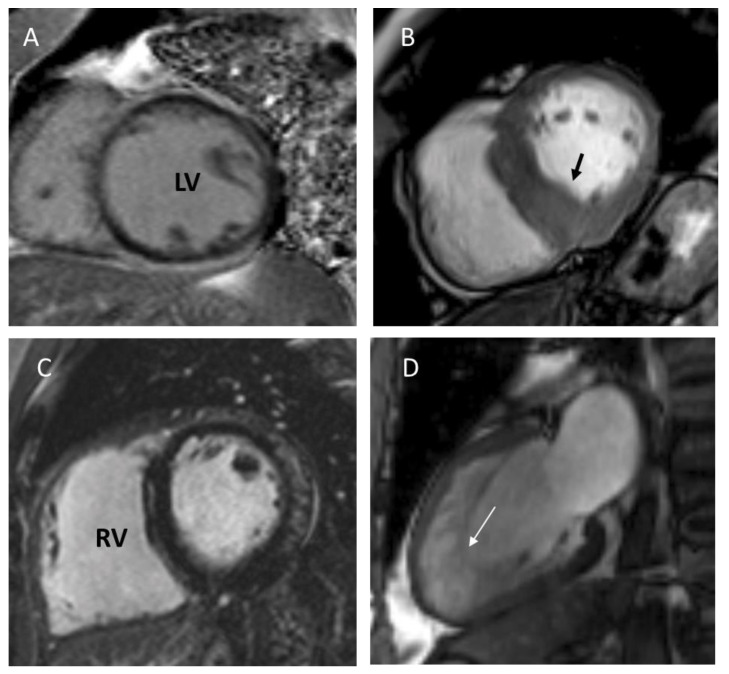
Cardiac magnetic resonance features of different cardiomyopathies. (**A**) Dilated cardiomyopathy: left ventricle (LV) dilation with thinning of the ventricular walls. (**B**) Hypertrophic cardiomyopathy: non-dilated LV with asymmetric hypertrophy (arrow). (**C**) Arrhythmogenic right ventricular (RV) cardiomyopathy: dilation and thinning of RV wall due to fibro adipose replacement. (**D**) LV noncompaction: increased ratio of non-compacted (arrow)/compacted layers of the LV wall. (Kindly provided by Dr André Carvalho, Radiology Department, Centro Hospitalar Universitário São João).

International echocardiographic criteria have been published to differentiate from trabeculation seen in normal individuals. Given that, LVNC is diagnosed, although with significant limitations, when the ratio between the two layers at end-diastole is greater than 2. In contrast, in a CMR, a ratio, in diastole, of noncompacted and compacted myocardium of at least 2.3 has been shown to be very reasonable at identifying pathological noncompaction, exemplified in Figure 2. Other measurements in CMR such as LV noncompacted mass can be useful to ascertain a diagnosis [45,52].

Some patients will end up developing LV dysfunction. Diastolic compromise has been reported as well due to abnormal relaxation, excessive trabeculation, or fibrosis. Arrhythmias, either ventricular tachyarrhythmias (more frequent) or atrial fibrillation, in association with the development of thrombi in the trabeculae are regarded as potential causes of morbidity [45]. All in all, sudden death accounts for half of the deaths attributed to LVNC [44].

Anticoagulation is recommended if the LV ejection fraction is under 40%, there is atrial fibrillation, or there is a history of thromboembolic events [53].

## 7. Genetic Testing

Nowadays, genetic testing is of paramount importance in the management of cardiomyopathies. First, certain variants might cause more severe phenotypes and have a higher risk of sudden death and thus must be addressed. Secondly, testing of family members will allow for the identification of at-risk individuals and provide them with proper vigilance [54,55].

Genetic testing in families should begin with the most affected individual, i.e., the one with the most severe manifestations or with the earliest onset of disease. Thereafter, the patient and their relatives should receive appropriate genetic counseling as well as cascade testing of other at-risk individuals. Nowadays, genetic testing comprises next-generation sequencing of multi-gene panels according to the individual’s phenotype. The timeframe has not been largely studied; however, it is generally considered reasonable at diagnosis [55].

Often, a single mutation can be responsible for any given cardiomyopathy. Nevertheless, compound heterozygous mutations have been reported in 5% of HCM cases and up to 20% of ACM cases [56].

Multi-gene panels offer a greater likelihood of finding the causal variant(s) but increase the chance of finding results of undetermined clinical significance. From single variant testing to whole exome sequencing, physicians benefit from a large array of possible options, and the selection of the specific type should be tailored to each situation. Not always will the addition of more genes prove to be beneficial. For example, when testing for DCM, even with a large panel containing more than 20 genes, a molecular diagnosis is achieved only in 10–40% of patients [57,58]. In contrast, almost 80% are pathogenic variants identified with current panels for HCM of only 2 genes (MYH7 and MYBPC3) [7,13,59].

According to the Heart Failure Society guidelines for cardiomyopathy genetic testing, LVNC should be seen more as a phenotype rather than a primary cardiomyopathy itself. Often, it appears in conjunction with all other primary cardiomyopathies. Hence genetic testing in LVNC should be directed by the cardiomyopathy phenotype presented in that case [55]. Indications for genetic testing according to each disease can be seen in Table 1. The most relevant genes are described in Table 2 [60].

## 8. Baseline Evaluation, Follow-Up, and Management

In cardiovascular genetics, the focus of diagnostic and treatment has moved from being solely on the index case and expanded to include the family. Often, probands when diagnosed are at late-stage disease, leaving few interventions available [55]. In addition, it has been shown that initiating therapy in asymptomatic individuals may improve outcomes due to heart failure [61,62].

Generally, a three-generation pedigree is recommended, with three main objectives: to determine if a familial predisposition is present, to ascertain the pattern of inheritance, and to identify at-risk individuals [16,55].

A detailed family inquiry, including cardiovascular symptoms such as shortness of breath, palpitations, syncope, and its relationship with exercise is advised. In addition, attributed diagnosis, namely, heart failure, and the history of prior procedures such as implantable cardioverter defibrillators, pacemakers, heart surgery, and transplantation with the respective age of onset are relevant. When it comes to sudden death, it should be especially relevant when under 40 years of age, in the context of single-vehicle accidents, drowning, or in infancy [55].

A clinical evaluation of first-degree relatives is recommended at baseline to classify them as clinically affected, clinically unaffected, or reportedly clinically unaffected (meaning asymptomatic but have not completed phenotype screening). This last category is relatively important because disease development is age dependent and requires successive assessments. If they have the phenotype of the disease, then expanded genetic testing could be required. Even individuals who test negative for a known familial variant are advised to be evaluated at baseline. However, if no signs or symptoms arise, there is no need for further follow-up [55].

Likewise, clinically unaffected individuals who are known to carry a family variant are advised to be regularly tested for the development of the phenotype. Clinical phenotype screening is comprised of four parts: medical history, physical examination, ECG, and a 2D echocardiogram, possibly with Doppler and CMR if the first imaging is equivocal [55,63].

Baseline phenotype screening for at-risk relatives also includes Holter monitoring, both in HCM and ACM, CK-MM (as in skeletal muscle creatinine kinase) in case of HCM and LVNC (to rule out syndromic and neuromuscular disease), and exercise treadmill testing if HCM. Lastly, in HCM and DCM, metabolic disease screening is recommended to exclude fatty acid oxidation defects and mitochondrial oxidative phosphorylation disorders [55].

The American Heart Failure Society has published guidelines applicable to first-degree relatives of individuals with established genetic cardiomyopathy [55]. They recommend clinical screening as described in the intervals detailed in Table 3.

## 9. Pregnancy

It is commonly known that pregnancy is associated with multiple hemodynamic changes, namely, significant increases in cardiac output, total plasma volume, and a reduction in peripheral resistance. Moreover, it determines a hypercoagulable state [64].

Usually, contraception is advised for females with cardiomyopathies at the onset of fertility, since that will allow for better planning of an adequate timeframe for the pregnancy. Factors such as disease stability and symptoms will be pondered in that decision. In addition, many drugs are risks for the unborn, requiring modifications to the therapeutic regimen [17]. Assessment of the necessity for certain preventative measures such as implantable cardioverter defibrillators is indicated and should be performed before pregnancy [65]. Additionally, due to their genetic etiology, appropriate preconception and prenatal genetic consults must be made available [65].

An initial risk assessment with the modified World Health Organization (WHO) classification of maternal cardiovascular risk is strongly suggested because a contraindication to pregnancy can be issued if Class IV and periodic outpatient cardiology evaluations are recommended in the following timelines: WHO class II—each trimester, class III—monthly or every 2 months [64,66].

Even women with established cardiomyopathies end up, in most cases, having no major events during pregnancy [17]. The limited number of maternal deaths related to cardiomyopathies primarily has occurred in women known to be of very high risk (for example with prior history of cardiac events or arrhythmia, poor functional class, or reduced systolic function) [67]. In addition, the worsening of clinical status due to being pregnant tends to happen in women already significantly symptomatic [68].

When it comes to FDM, the literature is very sparse. Few studies have been conducted, and often they are regarding idiopathic DCM. Nevertheless, the outcomes for genetic vs. idiopathic DCM are probably the same. In that case, asymptomatic and mildly symptomatic patients tend to have limited risk associated with pregnancy. Indicators to look for are severe LV dysfunction and poor New York Heart Association (NYHA) functional class [65]. So far, it is known that when DCM is diagnosed in the first trimester, worst outcomes are to be expected [69].

A 2022 retrospective, longitudinal study found that pregnancy did not accelerate disease progression in women carrying LMNA mutations, nor did it lead to worsening of electrical disease and ventricular arrhythmias (VAs), and it also did not increase the odds of needing a left ventricular assist device, a heart transplant, or even dying [70].

Peripartum cardiomyopathy (PPCM) is a diagnosis of exclusion. It can be understood as an idiopathic de novo cardiomyopathy with severe left ventricular systolic dysfunction in late pregnancy or the puerperium. PPCM can have similar characteristics with DCM and familial cases of PPCM have been reported. Both PPCM and pregnancy-associated cardiomyopathy can be an initial manifestation of FCM. The course of the disease can worsen; however, virtually half of the affected individuals will achieve normalized ventricular function in 2 years [65,71,72].

Lastly, regarding LVNC and ACM, current evidence is even scarcer. Of the case reports published about the latter in asymptomatic patients, no serious risk of disease progression with the pregnancy was found. It, most likely, poses a severe risk when associated with VAs or heart failure, though formal evidence is still lacking. When it comes to LVNC, due to its increased risk of thromboembolic events, anti-coagulation therapy has been conducted during pregnancy, yet without proper data [71].

## 10. Physical Activity and Athletes’ Recommendations

Exercise has always been associated with multiple health benefits. However, high-intensity exercise has been proven to be correlated with SCD events [73], which has led to generalized exercise restriction in patients with cardiomyopathies. HCM and ACM are the most frequent cardiomyopathies linked with SCD in the context of physical exertion [74].

Recently, new evidence has emerged suggesting that the relationship between physical activity and SCD could have been, previously, overestimated. For example, in individuals with HCM that participate in sports, defibrillators did not register a higher number of shocks than those implanted in patients not participating in sports [75]. In a cross-sectional study of patients with genetic HCM and LVH, exercise was even associated with better diastolic function without an increased risk of VAs [76].

Nowadays, it is reasonable that asymptomatic individuals carrying an HCM mutation, without any imaging finding of LVH, take part in competitive athletics without meaningful restriction [77,78]. Once LVH is found, caution is advised, and they should avoid high-intensity sports [77].

However, when it comes to ACM, the evidence seems to sustain a dose-dependent relationship between endurance training and the development of ACM. In a 2013 cross-sectional study, of 87 desmosome mutation carriers, those participating in endurance training and regular exercise were more likely to develop the phenotype at an earlier age than those who did not exercise or did it at a lower intensity. Patients in the highest quartile of exercise showed an odds ratio (OR) = 25.3, with a 95% confidence interval (CI) of 4.21–153. In addition, time free of VAs and heart failure was significantly higher in nonathletes [79]. Other studies have maintained that exercise might increase gene penetrance in ACM mutation carriers; thus, clinicians should warn adolescent and adult patients to refrain from competitive sports and high-intensity physical activity, keeping in mind the risk might be significantly lower for those without a family history [39].

When it comes to DCM, high-intensity and competitive sports are contraindicated in those carrying LMNA or FLNC mutations or with histories of cardiac arrest, unexplained syncope, LVEF < 45%, frequent VAs, or >20% LGE on CMR [78]. Besides those exceptions, low to moderate exercise may improve functional class [80].

LV hypertrabeculation is commonly found in athletes, with 8% of them meeting diagnostic criteria [81]. However, patients with high-risk genotypes (LMNA or FLNC mutations), those with LVEF < 40%, or those with frequent and/or complex VAs should refrain from high to very-high physical activity [78].

All in all, limited evidence has been produced to justify banning indiscriminately all individuals from participating in recreational sports. High-intensity exercise likely confers an increased risk of sudden death events in patients with a predisposition to developing arrhythmias. Nonetheless, a personalized decision based on appropriate testing is preferred, and low to moderate physical activity could be useful to prevent further health burdens attributed to sedentarism [74,78].

## 11. Sudden Death

As many as 30% of all sudden deaths are the first cardiac symptoms a patient may experience [82]. SCD can sometimes be the initial manifestation of familial cardiomyopathy. Both HCM and ACM have been reported as the leading causes of SCD. HCM has been cited to reach up to 36% of SCD [73]. Additionally, VAs are common in patients with genetic cardiomyopathies, increasing the chance of SCD. Timely risk stratification and implementation of preventative measures can significantly reduce morbidity and mortality. The main etiologies of death in familial cardiomyopathies include fatal arrhythmias, thromboembolic events, and heart failure.

In HCM, rates of sudden death are greater at younger ages and decrease into adulthood. The current state-of-the-art treatment has had a major impact on the number of those fatalities due to the usage of ICD devices, better advanced life support outcomes, and higher rates of heart transplantations [83]. To assess the risk of SCD, Holter ECG, echocardiogram, and exercise testing are important tools. The four main factors of concern are a family history of sudden death, personal history of inexplicable syncope, non-sustained ventricular tachycardia, and an LV wall thickness equal to or greater than 30 mm [17]. A 2014 study has subsequently proposed a formula to determine SCD risk at 5 years using these parameters [84]. Moreover, indicators including but not limited to LGE in CMR are promising predictors of overall disease outcomes and sudden death events [85].

In the case of DCM, SCD occurs in as many as 12% of patients, representing up to 30% of all mortality [86,87]. LMNA mutations have been emphasized as posing a greater risk of conduction disease and VAs. Hence a lower threshold for ICD implantation should be considered in those patients [88]. Phospholamban (PLN) mutation, filamin C (FLNC) gene truncating mutations, and a frameshift mutation of the BAG3 gene have also been recognized to cause higher rates of SCD [85]. Many imaging and electrophysiologic parameters have been shown to correlate with an augmented occurrence of arrhythmic outcomes and consequently SCD [85]. In a systematic review of almost 3000 patients, LGE was strongly and independently associated with VAs, increasing the risk four fold (OR = 4.3; 95%CI 3.3–5.8) [85].

ACM is associated with VAs and sudden death. A higher predisposition is seen in younger patients and those presenting with right ventricle dysfunction or syncope, ECG anomalies such as QRS fragmentation, or non-sustained ventricular tachycardia. Out of all possible measures, ICD implantation seems to be the most successful at preventing SCD [89]. Thus, effective selection of at-risk individuals is of utmost importance. The Task Force for ACM diagnosis recommends ICD in patients with previous episodes of sustained ventricular tachycardia or aborted SCD, while the Heart Rhythm Society also includes patients with a previous history of syncope and LV ejection fraction ≤ 35% [39,90].

In a study of LNVC patients, of those who died, half were from SCD. The main predictor of that occurrence was heart failure. Therefore, ICD implantation is indicated in those with LV ejection fraction ≤35%, NYHA functional class II or III, a family history of sudden death, a personal history of syncope, or the presence of nonsustained ventricular tachycardia [53].

Lastly, another factor that impacts the risk of SCD is the presence of multiple mutations. For example, triple sarcomeric mutations in HCM individuals conferred higher rates of SCD [91]. In addition, in a subset of patients with compound mutations related to ACM, more severe phenotypes were found, as well as more frequent sudden death events [92].

## 12. Conclusions

Although the management of genetic cardiomyopathies requires a high level of expertise, some key principles apply to all. First, the recognition of a familial predisposition is essential. Secondly, providing at-risk relatives with appropriate genetic testing and follow-up is another important step. Lastly, patients with an increased risk of sudden death must be identified and offered preventative options.

Due to their relative specificity, current recommendations tend to lack robust data. Some emerging areas of research are the relationship between genotype and phenotype, as well as their respective prognostic value.

## Figures and Tables

**Table 1:** Genetic testing recommendations according to an expert consensus Statement. Adapted from Wilde, A.A.M. (2022) [60].

HCM	All patients, and once a mutation is found, cascade testing of first-degree family members.
ACM	
DCM	Patients with positive family history.
	Patients presenting at younger age, with LV ejection fraction <35%, and a history of ventricular tachycardia or arrhythmia.
LVNC	Expert opinion recommends testing of patients and family members.
Children should be tested above 10–12 years old, or more prematurely if family history of early-onset disease.	

HCM—hypertrophic cardiomyopathy. DCM—dilated cardiomyopathy. ACM—arrhythmogenic cardiomyopathy. LVNC—left ventricular noncompaction.

**Table 2:** Frequent mutations associated with genetic cardiomyopathies. The most important genes in clinical practice are in bold. *Online Mendelian Inheritance in Man* numbers are indicated after each gene. Adapted from Judge, D.P (2008) [12] and Wilde, A.A.M (2022) [60].

		HCM	DCM	ACM	LVNC
Sarcomere	ACTC1—102540	X	X		X
	TNNT2—191045	X	X		X
	TNNI3—191044	X	X		
	MYH7—160760	X	X		X
	MYBPC3—600958	X	X		X
	TPM1—191010	X	X		
	MYL2—160781	X			
	MYL3—160790	X			
	TNNC1—191040	X	X		
	TTN—188840	X	X		X
	MYH6—160710	X	X		
Z-Disc	FLNC—102565	X	X	X	
	TCAP—604488	X	X		
	MYOZ2—605377	X	X		
	CSRP3—601225	X	X		
	ACTN2—102573	X	X		
	MYPN—608517		X		
Cytoskeleton	DYS—300377		X		
	DES—125660		X	X	X
	LDB3—605906	X	X		
	SGCD—601411		X		
	PDLIM3—605906		X		
	VCL—193065	X	X		
	CRYAB—123590		X		
Nuclear envelope	LMNA—150330		X		
	EMD—300384		X		
	ILK—602366		X	X	
	LAP2—150320		X		
	LAMA4—600133		X		
	TMEM43—605676			X	
Ion channel	SCN5A—600163		X		
	ABCC9—601439		X		
Mitochondria	FKRP—606596		X		
	tRNA		X		
	TAZ—300394		X		X
	ND1	X			
	FRDA—229300	X			
	ANT1—103220	X			
Transcription factor	EYA4—603550		X		
Sarcoplasmatic reticulum	PLN—172405	X	X	X	
	JPH2—605779	X	X		
Transmembrane	PSEN1—104311		X		
	PSEN2—600759		X		
Storage disease	PRKAG2—602743	X			
	LAMP2—309060	X			
	GLA—300644	X			
	GAA—606800	X			
Desmosome	PKP2—602861			X	
	DSP—125645			X	
	DSG2—125671			X	
	DSC2—125645			X	
	JUP—173325			X	
Growth factor	TGFB3—190230			X	
Other	PTPN11—176876	X			
	CAV3—601253	X			

HCM—hypertrophic cardiomyopathy. DCM—dilated cardiomyopathy. ACM—arrhythmogenic cardiomyopathy. LVNC—left ventricular noncompaction. ACTC1—cardiac actin 1. TNNT2—troponin T. TNNI3—cardiac troponin I. MYH7, MYH6—cardiac beta-myosin heavy chain. MYBPC3—cardiac myosin binding protein C. TPM1—alpha-tropomyosin. MYL2, MYL3—myosin light chains. TNNC1—cardiac troponin C. TTN—titin. TCAP—telethonin. CSRP3—cysteine- and glycine-rich protein-3. MYOZ2—myozenin-2. ACTN2—alpha-actinin-2. MYPN—myopalladin. DYS—dystrophin. DES—desmin. LDB3—LIM domain binding 3. SGCD—delta-sarcoglycan. PDLIM3—LIM domain protein 3. VCL—vinculin. CRYAB—crystallin alpha B. LMNA—lamin A/C. EMD—emerin. ILK—Integrin Linked Kinase. LAP2—Thymopoietin. LAMA4—Laminin Subunit Alpha 4. TMEM43—transmembrane protein 43. SCN5A—sodium voltage-gated channel alpha subunit 5. ABCC9—ATP Binding Cassette Subfamily C Member 9. FKRP—fukutin-related protein. tRNA—transfer RNAs. TAZ—tafazzin. ND1—NADH-ubiquinone oxidoreductase chain 1. FRDA—Friedreich’s Ataxia gene. ANT1—adenine nucleotide translocator 1. EYA4—EYA Transcriptional Coactivator And Phosphatase 4. PLN—phospholamban. JPH2—junctophilin 2. PSEN1—presenilin-1. PSEN2—presenilin-2. PRKAG2—Protein Kinase AMP-Activated Non-Catalytic Subunit Gamma 2. LAMP2—Lysosomal Associated Membrane Protein 2. GLA—Galactosidase Alpha. PKP2—plakophilin 2. DSP—desmoplakin. DSG2—desmoglein-2. DSC2—desmocollin 2. JUP—junction plakoglobin. TGFB3—transforming growth factor-beta. PTPN11—protein tyrosine phosphatase non-receptor type. CAV3—caveolin-3.

**Table 3:** Timeframes for the follow-up of first-degree relatives of individuals with a genetic cardiomyopathy. Adapted from Hershberger, R.E. (2018) [55].

	Infancy	Adolescence	Adulthood	Elderly
HCM	1–2 years	1–3 years	5 years	5 years
DCM			2–3 years	
ACM	5 years			3 years
LVNC	No relevant data were collected			

Elderly are considered above 50 years old. HCM—hypertrophic cardiomyopathy. DCM—dilated cardiomyopathy. ACM—arrhythmogenic cardiomyopathy. LVNC—left ventricular noncompaction.

## Data Availability

Not applicable.
